# Detecting the Pedestrian Shed and Walking Route Environment of Urban Parks with Open-Source Data: A Case Study in Nanjing, China

**DOI:** 10.3390/ijerph17134826

**Published:** 2020-07-04

**Authors:** Zhenqi Zhou, Zhen Xu

**Affiliations:** College of Landscape Architecture, Nanjing Forestry University, Nanjing 210037, China; ryanzhou@njfu.edu.cn

**Keywords:** walking route, urban park, pedestrian shed, pedestrian environment, open-source data

## Abstract

The propensity for visiting urban parks is affected by the park’s attractiveness and travel convenience, where walking provides the most basic and fair access. Walking routes from residences to parks, in terms of duration and perception, have received insufficient attention in the literature, particularly in the urban form context in China. Using the case study of Xuanwu Lake Park in Nanjing, we acquire walking routes from residences to the park through open-source data scraping in order to depict the pedestrian shed and pedestrian environment reasonably along these routes. The results show that the walking routes vary significantly with regards to distance, turns, street views, and so on. Proximity to urban parks, in terms of Euclidean distance, does not necessarily correspond to actual route distance, which may have a more direct influence on travel convenience and, hence, visiting propensity. Palpable differences in green visual ratio, image elements, and points of interest along these routes may also contribute to pedestrian environmental disparity. Analyzing data obtained from an online map provides a rapid and objective approach to detect pedestrian sheds and diagnose pedestrian environments, which can facilitate urban planners and policy makers in siting new parks and assessing the service capacity of parks.

## 1. Introduction

Visiting parks provides considerable health benefits, including supporting physical activity [1,2], reducing the risk of obesity [3], and improving the life expectancy [4] of citizens. Whether people walk to a park is usually affected by the park’s attractiveness and their walking experience [2,5]. The latter is associated with the walking distance to the park and relevant urban design features along the route, which have received insufficient attention, compared to the facilities and environments within parks. Environmental factors have been found to influence people’s walking choices around parks [6,7,8]. For example, routes to the park that cover long distances, potential traffic injury risks, and poor pedestrian environments could be substantial barriers for park visitors [2,9]. Furthermore, the walkability of streets near parks has been particularly correlated to pedestrian perceptions of safety [5]. Therefore, analysis of walking routes to parks provides a promising approach to understand the physical environment around parks, which may facilitate predicting public health benefits for people and environmental benefits for cities.

Previous proposals and practices, such as the Radburn Principle, Superblock, and Neighborhood Unit, have proved that healthy and walkable neighborhoods are key to maintaining livability in cities [10,11,12]. Updating these rules, Melbourne, Australia, launched the 20-minute Neighborhood Pilot Program in 2018 [13]. Shanghai, Jinan, and other cities in China have successively issued The Planning Guidance of 15-Minute Community-Life Circle since 2016 [14,15]. Parks are essential outdoor amenities in community-living circles, a 15-min walk to which is one of the crucial goals. A reasonable walking distance is necessary for daily park users [16]. Therefore, accurate measurement of the park’s pedestrian shed is essential. In earlier studies, most researchers have used the Euclidean distance buffer method to define a park’s service area, including the pedestrian shed as a circle around the park entrances [17]. For the simplicity of understanding and calculation, this method is still used in some planning and research approaches, even though non-straight line travel will be ignored in most cases [18]. Talen (2002) and Frank et al. (2005) adopted network analysis for further delineating a park’s service area. By connecting the discrete endpoints of paths that can be reached within a certain route distance, an irregular polygon was created as the pedestrian shed [19,20]. This method was more precise than the Euclidean distance buffer, even though some area within the polygon may still actually be inaccessible, in terms of distance, due to barriers of private land, water bodies, and so on. Alternatively, Oliver et al. (2007) assumed that most walks occur on public sidewalks or along roads, and proposed a line-based network buffer that defined areas near the centerline of routes as accessible [21]. Sandalack et al. (2013) also argued that the line-based network buffer method was more accurate, as it is closer to the actual environment available to pedestrians [22]. However, this method, which is based on GIS network analysis, depends on the availability of spatial data, making it quite expensive or even unfeasible for common researchers in countries with strict data control. Online map services, as potential open-source data, have provided opportunities to delineate pedestrian sheds. For instance, using the route navigation function of such services, researchers have been able to calculate the travel time and distance to certain facilities [23,24]. Compared to the traditional GIS software approach, mining online map services provides more precise measurements for better maintaining spatial information, as well as less time-consuming data preparation and non-frustrating data access, giving that any researcher can study it with only some coding skills.

Published evidence supports that walkable routes are not only affected by a reasonable distance but also the environmental quality [2,9,16]. In the last two decades, pedestrian environmental factors (e.g., pedestrian route directness, green visual ratio, and landscape features) and relevant audit tools (such as SPACES and PEDS) have received increasing attention in the urban planning and transportation fields, as well as in health-related studies [25,26,27,28,29]. Lee and Moudon (2006) found that walking was more sensitive to nuanced environmental characteristics compared to other transportation modes. Subsequently, Lee et al. (2015) used a pedestrian environmental audit tool and questionnaire survey research method to analyze the correlation between health-related satisfaction with the residential environment and pedestrian environment; they found out that safety, beauty, and functional aspects of the environment highly correlated with the satisfaction of residents [30]. These studies imply that fine-grained data and spatial unit analysis are critical for auditing pedestrian environments [31]. Open-source data provides a rough, yet rapid, approach to measure streets and neighborhood environments without tedious in-site investigation, as demonstrated in Rundle et al. (2011) [32] and Yang et al. (2019) [33]. However, most of the above studies have focused on the measurement of the pedestrian environment along streets, rather than potential or actual routes of travel by individuals, especially for park visitors. Dills et al. (2012) used an objective environmental auditing method to determine the influence of road walkability on park visiting, and found that traffic, beauty, safety, street maintenance, and neighborhood maintenance were positively related to park visiting [2]. With microscale variables such as pedestrian facilities, Rigolon et al. (2017) found that the walkability of routes to parks did contribute to the public health status of neighborhoods [5].

The goal of this paper is to provide a quantitative description of the walking route to a park using novel distance from home to the park and pedestrian environment measures. These objective measures use novel pedestrian shed auditing tools and scraped open-source data. The measures are summarized into specific indices that may be useful when delineating pedestrian pathways to parks. The indices are described in detail in the next section. While we use a specific park in this paper, the indices can be easily applied to any number of parks in any locale.

## 2. Materials and Methods

### 2.1. Case Study of the Park

Xuanwu Lake Park is located near the downtown of Nanjing, China, with the Purple Mountain to its east and the Ming City Wall to its west. As the largest free park in the city, it has been an important place for the daily recreation of citizens since the early 1900s. Xuanwu Lake Park has a total area of 502.08 ha (of which 378 ha is the lake), a perimeter of 10.79 km, and 26 entrances (scraped from Baidu Map in August 2019, and corrected by ground survey [34]).

### 2.2. Data Description

**Figure 1:**
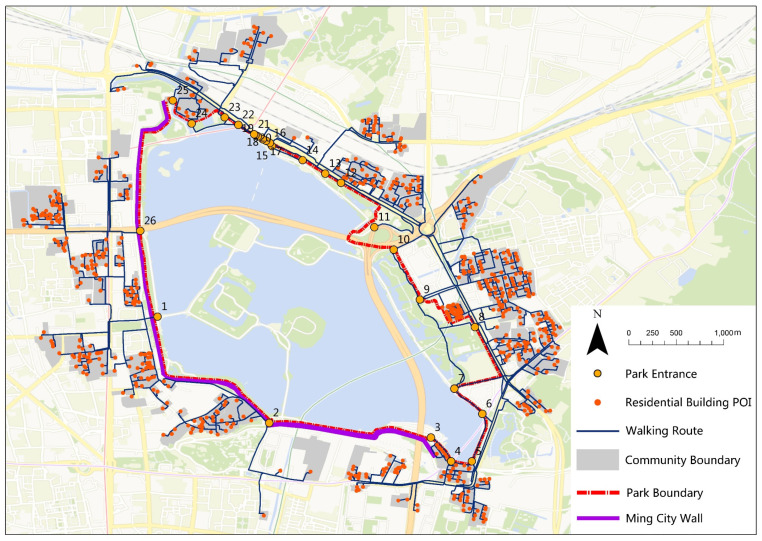
Fifteen-minute walking routes that define Xuanwu Lake Park’s pedestrian shed (base map from Tianditu Map).

Baidu Map (http://map.baidu.com) is a popular online map in China, which has been used to search for geographic data (such as POIs and travel routes) in recent studies [35]. We first acquired 664 residential building points of interest (POIs) and 100 community boundaries (scraped in August 2019) of surrounding areas of Xuanwu Lake Park from Baidu Map [34]. According to the policy of the 15-Minute Community-Life Circle, a 15-min walk to parks is important for residents in China, which corresponds to about a 1000-m distance. Coding with Python 3.7, we called the travel navigation function of Baidu Map to scrape 1554 recommended walking routes from residential buildings to the entrances of Xuanwu Lake Park, with a 15-min duration as the threshold (processed in August 2019). The recommending routes were taken as the shortest routes, according to the Application Programming Interface (API) of Baidu Map, which also included the information of route distance, duration, starting, and ending points [36]. Beside park entrances, residential buildings, and the walking routes connecting them (Figure 1), the data obtained in this study also included (1) population density data of Nanjing (100 × 100 m precision raster data in 2018), (2) street view photo data (15,528 photos, scraped in October 2019, from Baidu Map), and (3) all types of POIs around Xuanwu Lake Park (scraped in August 2019, including restaurants, hotels, markets, public facilities, scenic spots, recreation facilities, gyms, schools, cultural-related facilities, medical facilities, automobile services, traffic facilities, banks, residential areas, companies, and administrative machineries [37]).

### 2.3. Indices

The park’s pedestrian shed was delineated by walking routes presumably reached within 15 min, according to Baidu Map. By virtue of these walking routes, the service capacity and pedestrian environment were also analyzed. The former covers three indices of the service capacity: service POIs, service area, and service population, while the latter covers seven indices of the pedestrian environment: route distance, pedestrian route directness (PRD), the number of turns, the number of crossings, POI density along the route, green visual ratio, and image elements of street view photo (Table 1).

## 3. Results

### 3.1. Service Capacity and Pedestrian Environment at Park Level

Based on the methods of Euclidean distance buffer and walking route separately, the service capacity of the park was calculated with both 500-m and 1000-m thresholds (Table 2).

The service POIs determined by the route-based method were significantly less than those by Euclidean distance buffer method. Similarly, the service area and service population by the former method were less than those by the latter method. From the analysis of the PRD in the Euclidean distance buffer method, the average PRD (1.58) within 1000 m was smaller than the average PRD (1.96) within 500 m. This indicates that the closer the residential building POIs were to the park, the higher the PRD and the lower the walking permeability. Such differences may be attributed to the barriers, including the Ming City Wall, around the park. Once the surrounding permeability improves, more residents would have a shorter walk to the park, including those outside of the park’s pedestrian shed determined here.

In the range of the pedestrian shed, the average distance was 727 m, the average duration was 620 s, the average PRD value was 1.56, and the average number of turns was 4.63.

### 3.2. Service Capacity and Pedestrian Environment of Entrances

The population density, urban form, and environment around the park’s entrances varied significantly; however, relying on the Euclidean distance buffer method to measure the service capacity and pedestrian environment of entrances gave indistinguishable results. Therefore, combining a more accurate population density and route-based approach, the entrance service capacities and pedestrian environments can be made more refined and reliable.

#### 3.2.1. Service Capacity of Entrances

**Figure 4:**
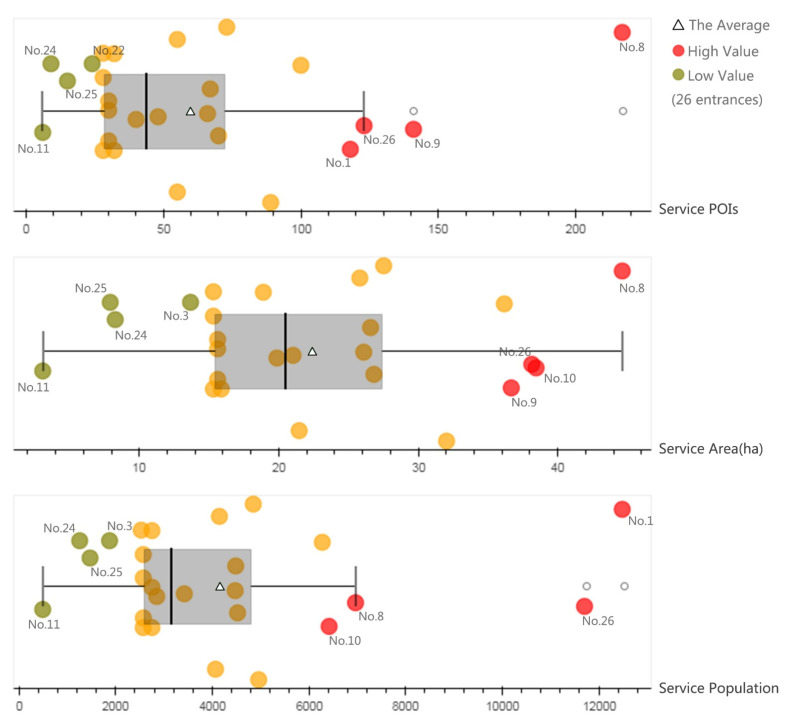
Service capacity of entrances.

The service POIs, area, and population across different entrances varied significantly, due to the extensive interface of Xuanwu Lake Park with various parts of the city (Figure 4). Entrances No. 8, No. 9, No. 26, and No. 1, which are located to the west and north of Xuanwu Lake Park, served more POIs: 217, 141, 123, and 118 POIs, respectively. Entrances No. 25, No. 24, and No. 22, located to the north of the park, served fewer POIs: 24, 15, and 9 POIs, respectively. Entrance No. 11 served the least POIs, just 6. The service area and service population shared similar trends. The average service POIs, area, and population per entrance were 60, 22.37 ha, and 4145, respectively.

**Figure 5:**
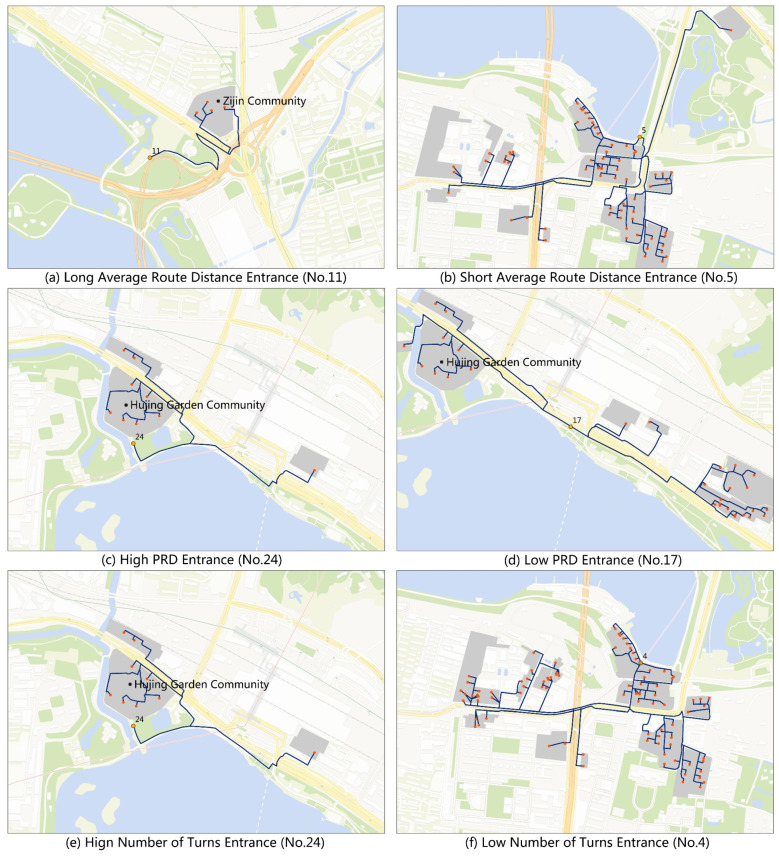
Cases of indices of entrances: (**a**) long average route distance entrance; (**b**) short average route distance entrance; (**c**) high PRD entrance; (**d**) low PRD entrance; (**e**) high number of turns entrance; and (**f**) low number of turns entrance (Base map from Tianditu Map).

Spatial analysis with GIS showed that entrance No. 11 only served Zijin Community (Figure 5a), and the average distance was 941 m, while entrance No. 10 could also serve Zijin Community, and the average distance was 614 m. Therefore, the service capacity of entrance No. 11 was low. Another case is entrance No. 24: pedestrians need to walk inside the park and through entrances No. 22 or No. 23 to arrive at their communities, as the south gate of Hujing Garden Community is closed (Figure 5c).

#### 3.2.2. Pedestrian Environment of Entrances

**Figure 6:**
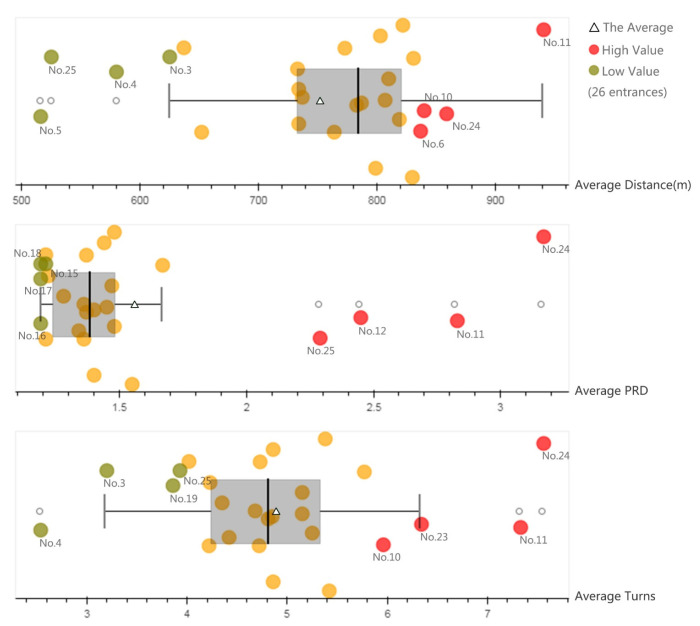
Pedestrian environment of entrances.

Average route distance: The average walking distance from residential buildings to entrances was 753 m (Figure 6), with the No. 11 and No. 24 routes ranked as the most extended distances: 941 m (Figure 5a) and 859 m, respectively. The distance of routes connecting entrances No. 25 and No. 5 were relatively short: 525 and 516 m (Figure 5b), respectively.

PRD: The average PRD from residential buildings to entrances was 1.56 (Figure 6). The PRDs of entrances No. 24 and No. 11 were as high as 3.17 and 2.83, respectively. The high PRD of entrance No. 24 can be attributed to the south gate of Hujing Garden Community being closed, which led to more roundabout routes (Figure 5c). The PRD of entrance No.11 was high mainly due to the tortuous road linking the entrance. The PRD of entrance No. 12 was high also (2.45), which can mainly be attributed to its proximity to the highway, resulting in a long walk for residents to travel across. The PRDs of Nos. 16–21 were as low as 1.20, indicating the more permeable street network near these entrances (Figure 5d).

The number of turns: The average number of turns along the routes was 4.91 (Figure 6). The turns relevant to entrances No. 24 and No. 11 were too high (7.56 times (Figure 5e), and 7.33 times, respectively), while entrances No. 4 and No. 3 had the lowest number of turns (2.54 times (Figure 6f) and 3.20 times, respectively), implying that residents near the two entrances could reach the park by a less roundabout route. The number of turns was somewhat correlated to PRD, where a more permeable urban area likely led to lower PRD and number of turns in relevant routes.

### 3.3. Pedestrian Environment of the Routes from Communities to the Park

**Figure 7:**
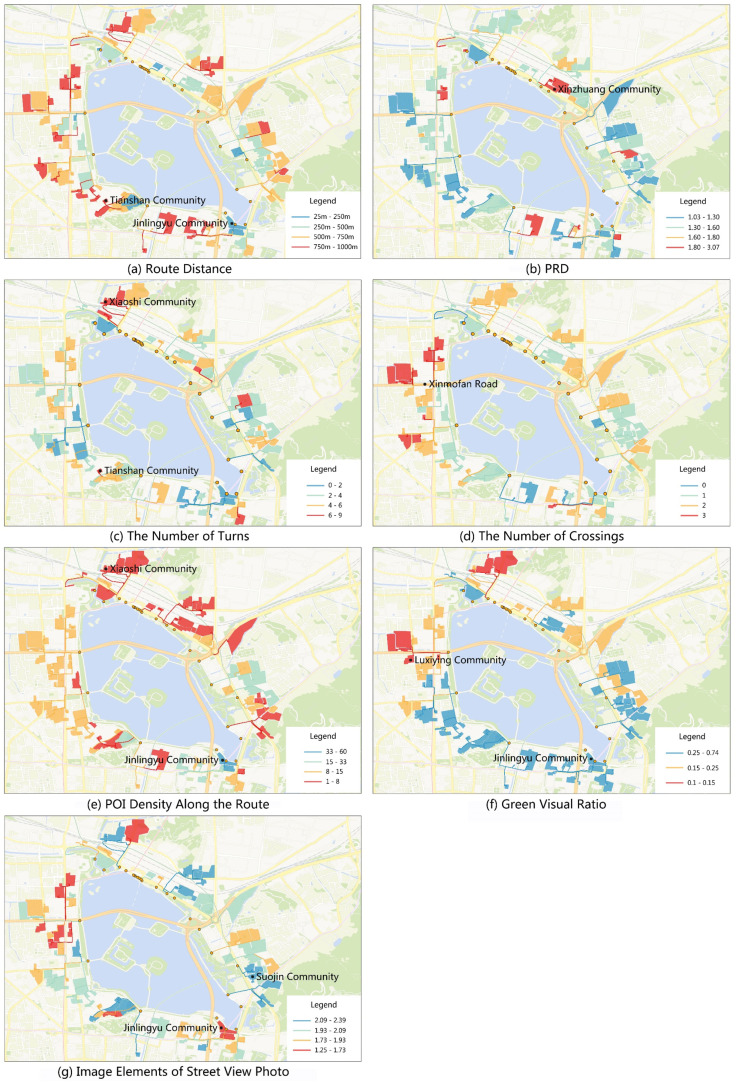
Pedestrian environment analysis of the routes from communities to the park: (**a**) route distance; (**b**) PRD; (**c**) the number of turns; (**d**) the number of crossings; (**e**) POI density along the route; (**f**) green visual ratio; and (**g**) image elements of street view photo (base map from Tianditu Map).

Taking the community boundary as the analysis unit, we selected the shortest route among residential building POIs in each community to analyze the pedestrian environment from communities to the park. The pedestrian environment of route analysis covered the criteria of route distance, PRD, the number of turns, the number of crossings, POI density along the route, green visual ratio, and image elements of street view photo, as shown in Figure 7.

Route distance: The average and median distances were 651 and 690 m, respectively. Eight routes were less than or equal to 250 m, 26 routes were less than or equal to 500 m, and 64 routes were less than or equal to 750 m. Walking routes exceeding 750 m accounted for 36% of the total. The shortest route—from Jinlingyu Community to entrance No. 4—was 25 m. Meanwhile, the longest route was 992 m—from Tianshan Community to entrance No. 1. Long-distance routes were mainly to the west and south of the park, especially those connecting to entrances No. 1, No. 26, and No. 2. Short-distance routes were mainly to the east of the park, especially those connecting to entrances No. 10, No. 7, and No. 5 (Figure 7a).

PRD: The average and median PRDs were 1.41 and 1.38, respectively. The PRDs of 34 routes were below 1.3. Communities with a low route PRD were mainly to the southeast and southwest of the park (Figure 7b). The PRDs of nineteen routes was above 1.6, and the PRDs of 12 routes was above 1.8. There were 23 routes longer than 750 m with PRD exceeding 1.3, of which the PRDs of eight routes was above 1.6. The long route distance and high PRD meant that the permeability between the residence and the park was low. The highest PRD was 3.07, for the route connecting Xinzhuang Community to entrance No. 13. Roadways, water bodies, closed communities, and the Ming City Wall were the main factors causing high PRD.

The number of turns: The average and median turn times were 4.08 and 4.00, respectively, for which 20 communities needed to turn more than six times. The residents of Xiaoshi Community and Tianshan Community needed to walk to the park with eight and nine turns along their routes, respectively. The number of turns in the communities connected to entrance No. 23 was high, with an average of 6.17 (Figure 7c).

The number of crossings: The average and median crossing times were 1.58 and 2.00, respectively. The residents of six communities did not need to cross the road to visit the park, while the residents of 43 communities needed to cross the road once and residents of 38 communities needed to cross the road twice. Residents of 13 communities needed to cross the road three times to reach the park, mainly via entrance No. 26 (Figure 7d), which connected to the main thoroughfare of Xinmofan Road.

**Figure 8:**
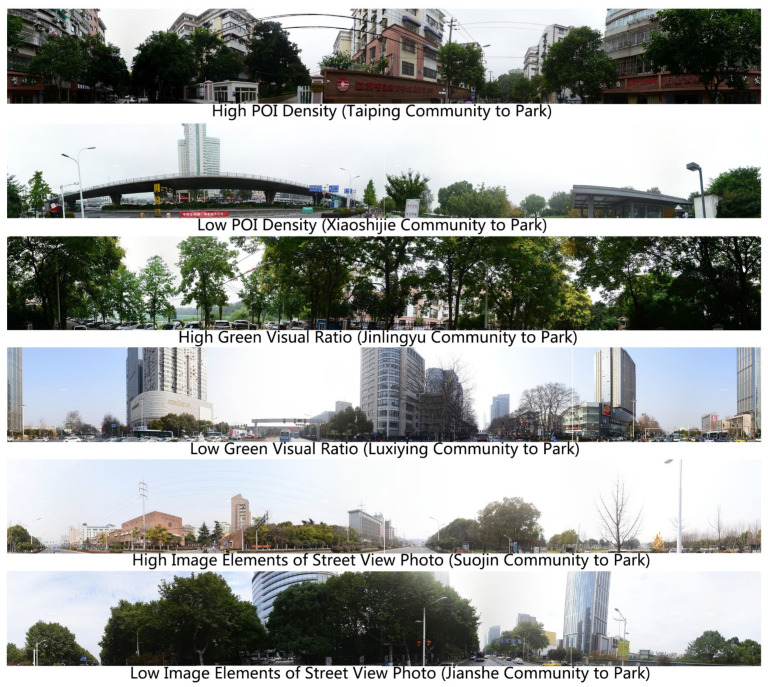
Street view photos of pedestrian environments.

POI density along the route: The average and median of POI densities along the route were 11 and 9, respectively, being smaller than or equal to 8 in 36 communities, lower than or equal to 15 in 86 communities, and higher than 33 in two communities. The POI density of the route from Jinlingyu Community to entrance No. 4 was the highest at 60 (i.e., there were 60 POIs along the route per 100 m). The route connecting Xiaoshijie Community and entrance No. 23 had the lowest POI density, only 1 (Figure 8). The routes connecting to the northeast and southwest sides of the study area generally had a low POI density; meanwhile, the routes connecting to entrances No. 9, No. 8, and No. 4 had a high POI density (Figure 7e).

Green visual ratio: The average and median green visual ratios were 29% and 30%, respectively. The green visual ratio of 88 routes was no less than 15%, while that of 60 routes was no less than 25%. The route with the highest green visual ratio (74%) was from Jinlingyu Community to entrance No. 4, whereas the route with the lowest green visual ratio (9%) was from Luxiying Community to entrance No. 26 (Figure 8). The green visual ratio of the routes around Xuanwu Lake Park are quite high, except in the routes connecting to entrances No. 23 and No. 26 (Figure 7f).

Image elements of street view photo: The average and median number of image elements along all the routes were 1.97 and 1.99, respectively. In terms of image element number, the value in ten routes was less than 1.73; in 34 routes, less than 1.93; and, in 24 routes, higher than 2.09. The route from Suojin Community to entrance No. 7 had the most image elements (2.4) (Figure 8), while the route with the least image elements (1.25) was from Jinlingyu Community to entrance No.4. The image elements of the routes connected to entrances No. 2, No. 23, No. 13, No. 8, and No. 7 were high, while the routes connected to entrance No.26 had few image elements (Figure 7g).

## 4. Discussion

Using open-source data from Baidu Map in this study, we analyzed the service capacity and pedestrian environment in Xuanwu Lake Park’s pedestrian shed at three levels: the entire park, the park entrances, and routes from communities to the park.

The measurement of the park’s pedestrian shed was based on the route-based method, involving three aspects—the residential building POIs, the community boundaries around the park, and the routes connecting the entrances and the residential building POIs—which was more suitable for the Chinese ubiquitous gated community pattern. Compared with previous studies, this method takes advantage of the online map service, the reliability of which is bolstered by the feedback of numerous users. This helps researchers to reduce their reliance on traditionally official data, hence saving time in data acquisition and field investigation [46].

In previous studies, the convenience, safety, and beauty of walking routes have been shown to directly affect resident’s willingness to walk [27,29,42]. For example, Jiao et al. (2017) investigated the pedestrian environment near subway stations in Shanghai. They found that the pedestrian route directness, pedestrian safety, and connectivity were highly correlated with overall pedestrian satisfaction [42]. Giles and Donovan (2002) found that the aesthetic and functional aspects of a neighborhood environment were positively related to walking [47]. An index in the Path Environment Audit Tool (PEAT) indicated that when there were shops near the walking route, pedestrian walking is promoted [48]. In this study, the route networks, visual openness, and pedestrian environments differed around the park. The seven indices used may facilitate the rapid and straightforward retrieval of pedestrian environments and pinpoint certain problems, such that planners can make targeted designs for better pedestrian environments.

Nanjing City has a dense population and buildings, which make it challenging to add new parks. To benefit more residents, it is therefore necessary to expand the capacity of the existing parks. Increasing the number of park entrances is a practical and easily implementable measure, but there may be some limitations in the case of Xuanwu Lake Park. Half of the park’s boundary is the Ming City Wall, which is protected as a location of cultural heritage. In 2016, the Central Urban Work Conference and the Ministry of Construction proposed a ban on constructing gated communities, as well as gradually opened existing gated communities, providing a promising vision for changing the low-permeability urban form in Chinese cities. Under this policy framework, improving the pedestrian penetration of the surrounding environment is a softer and more pragmatic approach. In this case, the service area of entrance No. 24 was limited, due to the closed south gate of Hujing Garden Community. According to the above analysis, opening this community gate would curtail travel time to the park from seven residential buildings to within 15 min (1000 m), with the average walking distance decreasing from 859 to 448 m, and the average PRD dropping from 3.17 to 1.31.

In this study, we detected the park’s pedestrian shed based on the route-based method and audited the pedestrian environment with open-source data. The measurement is simple to apply, which may provide reference for assessing the park’s service extent and its potential capacity, as well as determining the siting of new parks.

There are some limitations to this study. First, it focused on the walking routes from residential buildings to the park, while ignoring users departing from the bus or metro stations and workplaces. Second, due to the fixed and limited data of the online map service used, it was difficult to evaluate some micro-environmental factors (e.g., pavement condition of the walking road and air quality) and immediate environmental factors (e.g., temporary obstacles and building construction) [49]. More research into street features that promote walking to parks is expected to refine this method in the future.

## 5. Conclusions

A walking route is an intermediary for understanding the mobility of urban residents and the physical environment. This study provides a perspective for understanding the walkable routes from communities to parks. First, the route networks, visual openness, and pedestrian environments differ around the urban park considered. Second, this study indicates the remarkable potential to accommodate more walking with landscape management, which may be supported by the policy of Enhancing City Blocks issued in 2016 in China. Scraped geographic data from an online map provided a rapid and simple approach to detect pedestrian sheds and diagnose pedestrian environments, which can help urban planners and policymakers to assess the service extent and potential capacity of parks, as well as to determine the siting of new parks. Comprehensive tactics on the routes to parks, such as setting up more park entrances, shortening the walking distance by opening gated communities, and improving the pedestrian environment, are suggested to support more residents in walking to parks. The tactics basically consist of urban design, including enhancing permeability both visually and physically.

## 6. Patents

1. Computer software copyright: Peripheral walking routes extractor software [abbreviation: Lines] V1.0
2. Computer software copyright: POI extractor software [abbreviation: POIs] V1.0
3. Computer software copyright: AOI extractor software [abbreviation: AOIs] V1.0
4. Computer software copyright: Walking simulation trip generator software [abbreviation: Walking] V1.0
5. Computer software copyright: Street view map extractor software [abbreviation: StreetPictures] V1.0
6. Computer software copyright: Image green vision extractor software [abbreviation: GreenRate] V1.0

## Figures and Tables

**Figure 2:**
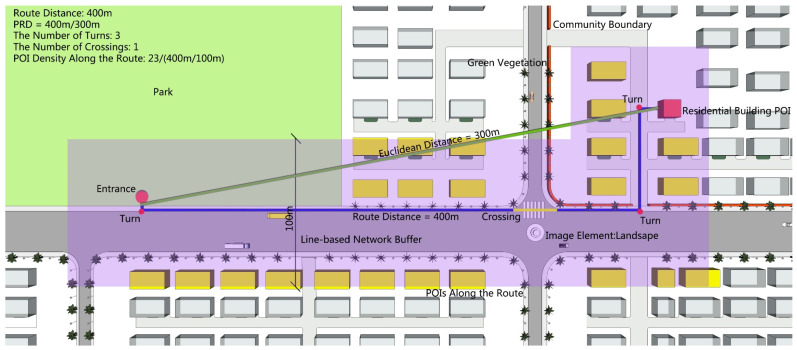
Route-based indices of the service capacity and pedestrian environment.

**Figure 3:**
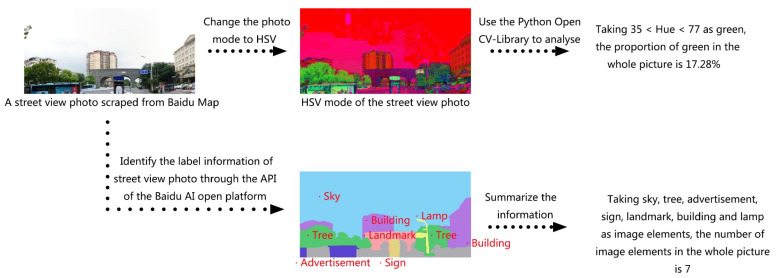
Indices diagram of green visual ratio and image elements of street view photo.

**Table 1:** Indices relevant to the walking routes to the park.

Index	Definition	Comment
Service POIs	The number of residential building POIs within pedestrian shed of the whole park or a specific entrance (Figure 1).	Describes the amount of residential buildings that the park (or its entrances) can serve [23].
Service area	The total area of communities within the pedestrian shed of the whole park or a specific entrance (Figure 1).	Describes the area that Xuanwu Lake Park (or its entrances) can serve [38].
Service population	The population within serviced communities.	Describe how many residents Xuanwu Lake Park (or its entrances) can serve [38].
Route distance	Distance of the route from residential building POIs to a park entrance (Figure 2).	Describes the distance of walking routes based on Baidu Map. These routes were derived from the travel navigation function of Baidu Map, and were the shortest routes in the walking algorithm of the Baidu Map API [36].
Pedestrian route directness (PRD)	The ratio of route distance to Euclidean distance (always greater than or equal to 1), reflects the degree of pedestrian route directness (Figure 2).	When the block scale is suitable and the road network is porous, the PRD is relatively low. In some western cities, the PRD in well-connected grid areas is generally less than 1.3. The sectors with low pedestrian permeability (more significant than 1.6) tend to be suburbs dominated by end roads [25,39]. The higher the PRD of a route, the more turns it has; however, the two are not linearly correlated [40].
The number of turns	The number of turns along a walking route (Figure 2).	To reflect the smoothness of walking, the number of times pedestrians need to turn to cope with the road conditions. This affects the wayfinding performance of people [41]. After a ground survey, the number of turns were found to be consistent with the actual direction changes.
The number of crossings	The number of crossings a walking route would traverse (Figure 2).	To reflect the smoothness of walking, the perceived safety of pedestrians, and the degree of traffic risk. Crossings mean to cross the road or sidewalk [42]. Considering the ground survey, the number of crossings were consistent with the actual crossing times of pedestrians.
POI density along the route	POI density along the route is measured by the ratio of the number of POIs in the 100 m line-based network buffer per 100 m distance (Figure 2).	In the walking route, the more POIs along the street, the higher the street safety and vitality [43].
Green visual ratio	The green visual ratio was obtained by analyzing the hue of street view photos through the Python OpenCV Library. The average value was taken as the green visual ratio of a route (Figure 3).	This refers to the proportion of green vegetation seen by pedestrians, which emphasizes the three-dimensional visual effect and represents a higher level of urban greening. When the green visual ratio is higher than 25%, people will feel better about the surrounding environment. When it is less than 15%, people’s perception of green quantity is poor [26,44].
Image elements of street view photo	Scenery such as sky, buildings, water, landmarks, natural scenery, railings, seats, street lamps, signs, and so on, are classified as image elements by identifying the label information of street view photos through the API of the Baidu AI Open Platform [45]. In a route, the ratio of total image elements to the number of street view photos was taken as the image elements of street view photo (Figure 3).	In the walking route, the landscape (such as sky, trees, and landmarks) and facilities (such as railings, seats, and streetlights) can improve the pedestrian walking experience [27]. The more identifying tag elements, the more image elements there are suitable for walking.

**Table 2:** Different methods for determining the pedestrian shed of Xuanwu Lake Park, in terms of service points of interest (POIs), area, population, and pedestrian route directness (PRD).

	Euclidean Distance Buffer Method				Route-Based Method			
Distance	Service POIs	Service Area (ha)	Service Population	Average PRD	Service POIs	Service Area (ha)	Service Population	Average PRD
500 m	276	54.23	8960	1.96	153	23.93	4029	1.57
1000 m	1148	379.59	87,505	1.58	664	206.88	44,540	1.56
